# Complete Genome Sequence of *Gordonia* sp. 135, a Promising Dibenzothiophene- and Hydrocarbon-Degrading Strain

**DOI:** 10.1128/MRA.01450-19

**Published:** 2020-01-09

**Authors:** Yanina Delegan, Leonid Valentovich, Anna Vetrova, Ekaterina Frantsuzova, Yulia Kocharovskaya

**Affiliations:** a Institute of Biochemistry and Physiology of Microorganisms, Pushchino, Moscow Region, Russian Federation; b Federal Research Center, Pushchino Scientific Center for Biological Research of the Russian Academy of Sciences, Pushchino, Moscow Region, Russian Federation; c Institute of Microbiology of the National Academy of Sciences of Belarus, Minsk, Belarus; d Kuban State University, Krasnodar, Russia; e Tula State University, Tula, Russia; Broad Institute

## Abstract

*Gordonia* sp. strain 135 is a promising dibenzothiophene-desulfurizing and hydrocarbon-degrading bacterium. It can utilize dibenzothiophene as the sole sulfur source. The genome of strain 135 was completely sequenced; it consists of a 5,039,827-bp circular chromosome and a 164,963-bp circular plasmid.

## ANNOUNCEMENT

*Gordonia* bacteria encompass a rich reservoir of metabolic diversity, which makes them attractive candidates for crude oil bioremediation (1). Most of the species present in this genus are capable of utilizing hydrocarbons and sulfur-containing organic compounds such as dibenzothiophene (DBT) (2, 3). The strain *Gordonia* sp. 135 was isolated from oil-polluted soil (in Moscow, Russia). It is capable of crude oil and DBT degradation (strain certificate VKM Ac-2849D). For long-term storage, the strain was kept in glycerol (40%) stocks at −70°С. For short-term maintenance, the strain was cultured on lysogeny broth agar plates at 27°C.

Genomic DNA was isolated from a fresh culture biomass (a colony) of *Gordonia* sp. 135 grown on LB agar using a DNeasy blood and tissue kit (catalog number 69506; Qiagen). Sequencing was performed using a MinION sequencer with the flow cell FLO-MIN106 (Oxford Nanopore Technologies [ONT]) (BioSpark, Moscow, Russia). A library was prepared with a rapid sequencing kit (catalog number SQK-RAD004). Guppy (version 3.2.4) software was used for base calling, which yielded a total of 100 Mbp distributed in 29,042 reads with a Q score of >10. Additionally, the same DNA sample was sequenced with an Illumina NovaSeq 6000 platform using an S2 reagent kit (catalog number 20012861; 2 × 100 bp). A paired-end library was prepared with the Kapa HyperPlus kit (Roche). The Illumina and Nanopore reads were used for hybrid assembly with SPAdes (version 3.13.1) (4). The Nanopore reads were assembled into 4 contigs using Flye assembler version 2.6 (5). Next, SPAdes contigs were combined into two replicons with the help of Flye data which were used as reference sequences. The Illumina reads were used to correct Nanopore or assembly errors using Bowtie2 version 2.3.5.1 (6) and Pilon version 1.23 (7) software. Default parameters were used for all software.

The *Gordonia* sp. 135 genome consists of a 5,039,827-bp circular chromosome (GC content, 67.44%) and a 164,963-bp circular plasmid, namely, pG135 (GC content, 64.58%). Chromosome and plasmid circularization was specified by end overlapping. The closest relative of the strain 135 is Gordonia alkanivorans YC-RL2 (GenBank accession number CP027114). The ANI value was calculated using EzBio Cloud ANI calculator (8). The average nucleotide identity (ANI) value of chromosomes is 97.45%. The plasmid pG135 is related to pKB1 from G. westfalica Kb1 (NC_005307) (91.31%) and pYYC01 from *G. alkanivorans* YC-RL2 (CP027115) (92.08%).

The strain 135 genome was annotated with the NCBI Prokaryotic Genome Annotation Pipeline (PGAP) version 4.6 (9). The chromosome contained 4,492 coding sequences, 4 rRNA clusters (5S, 16S, and 23S), and 51 tRNAs. The genome of *Gordonia* sp. 135 has no alkane hydroxylase gene (*alkB*) but does have 3 copies of cytochrome P450 hydroxylase (CYP153). So, we reasoned that alkane-degrading capabilities of the strain are associated mainly with the cytochrome P450 hydroxylase activity (10). The genes that may be responsible for DBT degradation were also detected.

The antiSMASH search for secondary metabolite clusters found 15 clusters on the chromosome, including clusters of ectoine and carotenoid production (11). The genome sequence data of *Gordonia* sp. 135 will enhance our understanding of the metabolic capabilities of *Gordonia* strains.

### Data availability.

This genome project has been deposited at GenBank under the accession numbers NZ_CP046257 for the chromosome and NZ_CP046258 for the plasmid and under BioSample number SAMN13326465, BioProject number PRJNA590456, and SRA accession number PRJNA590456.
